# Fat Chance: The Rejuvenation of Irradiated Skin

**DOI:** 10.1097/GOX.0000000000002092

**Published:** 2019-02-05

**Authors:** Mimi R. Borrelli, Ronak A. Patel, Jan Sokol, Dung Nguyen, Arash Momeni, Michael T. Longaker, Derrick C. Wan

**Affiliations:** From the * Hagey Laboratory for Pediatric Regenerative Medicine, Department of Surgery, Plastic and Reconstructive Surgery Division, Stanford University School of Medicine, Stanford, Calif.; † Institute for Stem Cell Biology and Regenerative Medicine, Stanford University, Stanford, Calif.

## Abstract

Radiotherapy (RT) helps cure and palliate thousands of patients with a range of malignant diseases. A major drawback, however, is the collateral damage done to tissues surrounding the tumor in the radiation field. The skin and subcutaneous tissue are among the most severely affected regions. Immediately following RT, the skin may be inflamed, hyperemic, and can form ulcers. With time, the dermis becomes progressively indurated. These acute and chronic changes cause substantial patient morbidity, yet there are few effective treatment modalities able to reduce radiodermatitis. Fat grafting is increasingly recognized as a tool able to reverse the fibrotic skin changes and rejuvenate the irradiated skin. This review outlines the current progress toward describing and understanding the cellular and molecular effects of fat grafting in irradiated skin. Identification of the key factors involved in the pathophysiology of fibrosis following RT will inform therapeutic interventions to enhance its beneficial effects.

## INTRODUCTION

The number of individuals affected by cancer in the United States is steadily increasing. In 2016, more than 15.5 million individuals had a current or previous diagnosis of cancer, and this is predicted to rise to 20.3 million by 2026.^1^ Operative tumor resection remains the first-line-treatment modality for many of these malignancies, but radiation therapy or “radiotherapy” (RT) is often used either exclusively, or in conjunction with chemotherapy and/or surgery. RT involves the projection of high-energy photons toward a tumor to damage the DNA of malignant cells, destroy their replicative ability, and ultimately cause their death. Although RT is extremely effective at shrinking tumor size and reducing local recurrence,^2–4^ it also causes a number of unwanted and long-term sequelae. One significant side-effect is the collateral damage done to healthy tissues in the radiation field.

The skin and subcutaneous tissue are among the most severely affected tissues during RT. The skin is vulnerable to damage during the treatment of breast cancer, head and neck cancer, and anal cancer, where skin-sparing techniques for delivering RT are not yet possible. Additionally, the high proliferative capacity and oxygenation requirements of basal epidermal cells makes them very radiosensitive.^5^ Radiation injury to the skin, also called radiodermatitis, occurs in over 90% of patients receiving RT for cancer.^6^ Classically, radiodermatitis is divided into the effects that occur in the acute phase following radiation exposure, and those which are evident after prolonged periods of time.^7^ The consequences of radiodermatitis can be profound; chronic soft-tissue fibrosis can significantly alter tissue form and function, which can significantly impact quality of life.

The histological effects of radiation-induced skin damage have long been described in the literature, but the pathogenesis driving these changes is less well understood. Additionally, while significant advances have been made towards the therapeutic delivery of RT, the treatment of radiodermatitis is underdeveloped. From a clinical viewpoint, radiodermatitis has been considered progressive, irreversible, and intractable. Recently, however, there has been increased focus on the use of autologous fat grafting (AFG) to rejuvenate and reverse the histological changes seen in radiodermatitis. Rigotti et al.^8^ were the first to demonstrate the beneficial effects of fat grafting in irradiated skin. Their observations have since been widely replicated and the mechanisms by which the grafted fat rejuvenates the skin remains an area of active research. Identifying the key factors that drive fibrotic skin damage following RT, and its mitigation by AFG, will highlight opportunities to enhance these restorative effects. This review outlines the current understanding of radiation-induced dermatitis, the therapeutic effects of AFG in the context of radiodermatitis, and the challenges facing this emerging treatment modality.

## RADIODERMATITIS

### Acute Effects of Radiotherapy

The early symptoms of radiation-induced skin damage include pigment alterations, erythema, edema, desquamation, ulceration, and loss of skin elasticity. On a cellular and molecular level, exposure to radiation initiates a number of cytokine cascades. Reactive oxygen species are also generated, and free radicals are released, causing irreversible breaks in double stranded nuclear and mitochondrial DNA. This process changes cellular function and induces apoptosis.^9^ Hair follicle stem cells and basal keratinocytes are highly proliferative and are especially vulnerable to the acute effects of RT, and their destruction impairs the self-renewing abilities of the skin.^7,10^ Damage to resident fibroblasts, endothelial cells, and epidermal cells causes them to release a number of pro-inflammatory growth factors, chemokines, and cytokines, including transforming growth factor beta 1 (TGF-β1), tumor necrosis factor alpha (TNF-α), interleukins (IL-1 and IL-6), basic fibroblast growth factor (bFGF), insulin-like growth factor-1 (IGF-1), and platelet-derived growth factor.^7,11,12^ These molecular signals activate the coagulation system and cause inflammation, tissue remodeling, and epithelial regeneration.

**Fig. 1.**
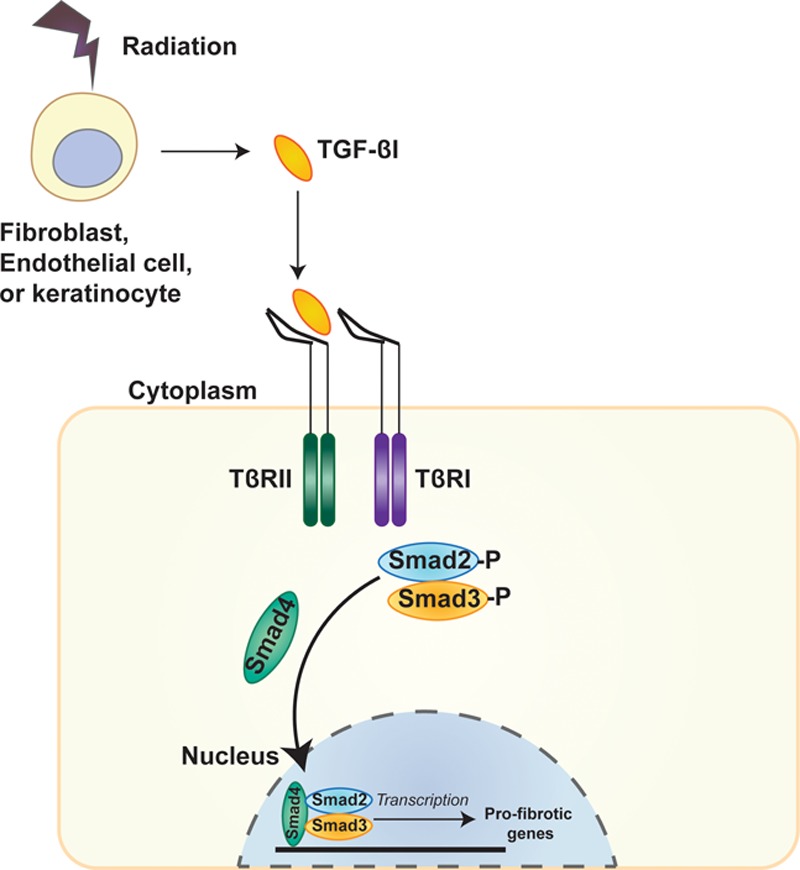
The TGF-β/Smad3 pathway: radiation damage results in TGF-β1 release from endothelial cells, fibroblasts, and keratinocytes. TGF-β1 binds the TβRII, which becomes phosphorylated and recruits the TβRI receptor. TβRI then phosphorylates the receptor associated Smads, Smad2, and Smad3, which bind Smad4, the common mediator. The receptor Smads and the common mediator form a complex, which translocates to the nucleus and acts as a transcription factor for a number of pro-fibrotic genes.

TGF-β1 is the principle growth factor/cytokine involved in radiodermatitis and is produced in abundance by skin fibroblasts, endothelial and keratinocytes upon exposure to radiation.^13^ Within hours, increased TGF-β1 levels are found in irradiated human, porcine, and mouse skin.^13–16^ TGF-β1 binds TβRI and TβRII—the transmembrane serine and threonine kinase receptors—which activates the intracellular signaling pathway mediated by the Smad proteins (Fig. 1). The receptor-associated Smads, Smad2, and Smad3 become activated/phosphorylated and heterodimerize with Smad4, the common mediator. Smad4 together with the receptor Smads, forms a complex which translocates to the nucleus and acts as a transcription factor for a number of profibrotic genes.^17–19^ Radiation-induced epidermal and dermal thickening in mice is correlated with an upregulation of the TGF-β/Smad3 fibrotic pathway.^20,21^ Additionally, wounds made in the skin of irradiated mice lacking Smad3 have fewer fibroblasts and myofibroblasts, less prominent but more organized collagen, and are less inflammatory than the irradiated skin of wild-type mice.^22,23^

One of the main roles of TGF-β1 is homeostasis of the extracellular matrix (ECM). TGF-β1 stimulation leads to increased production of ECM proteins, decreased production of matrix degrading proteases, and increased production of the inhibitors of these proteases. TGF-β1 also promotes the differentiation of fibroblasts into myofibroblasts. Myofibroblasts are “activated” fibroblasts with high proliferative capabilities and the ability to secrete copious amounts of fibrous matrix including collagen, fibronectin, and proteoglycans in response to TGF-β1. TGF-β1 further regulates the release of bFGF, TNF-α, and IL-1 by modulating their release and/or their production in endothelial cells and smooth muscle cells.^11,14,15^

The dermis is rich in blood vessels, which are also susceptible to damage from radiation. The smaller arterioles and capillaries are the most severely affected. In the acute phase, radiation increases the permeability of blood vessels, leading to tissue edema and intravascular thrombosis and fibrosis.^24^ Fibrin plugs form in blood vessels within hours of exposure, which obliterate blood flow leading to tissue hypoperfusion, ischemia, and ultimately atrophy.^11^ A significant reduction of blood flow is evident in irradiated, compared with nonirradiated, human and mouse skin.^21,25,26^

### Chronic Effects of Radiotherapy

Chronic radiodermatitis is marked by significant induration of the dermis and subcutaneous tissue, telangiectasia, and hyalinization of collagen of the reticular dermis. The epidermis may be hyperplastic or become atrophic, ulcerated, and necrotic, or develop skin tumors.^27,28^ These chronic fibrotic changes are the result of the continued release of cytokines and growth factors, which remain elevated for extensive periods of time even after the radiation source is removed. Biopsies from breast cancer patients treated with adjuvant RT may exhibit upregulated gene expression for collagen types I and III and TGF-β1 up to 20 years after RT.^29^ In porcine and mouse skin, radiation-induced elevated TGF-β1 levels remain high for up to 12 months after radiation and are localized to myofibroblasts, endothelial cells, and the collagen matrix.^15,30^

The continued abnormal production of stimulating cytokines and growth factors likely mediates a chronic state of cellular activation. Histological studies in mice indicate there is prolonged fibroblast proliferation^27^ and progressive ECM deposition with increasing time postirradiation. There is sustained myofibroblast activity, and the usual regulatory feedback mechanisms controlling myofibroblast activation and ECM production and degradation are disrupted.^14^

Vascular density is also decreased in irradiated, chronically injured skin.^20^ There is a dose-dependent reduction in the number of capillaries, the regularity of their distribution, and an increase in pericapillary fibrosis.^31^ The relative hypoxia stimulates the budding of irregular and easily occluded capillaries. The compromised tissue perfusion and resultant tissue hypoxia further augments the action of TGF-β1 and stimulates fibroblasts to proliferate and increase their expression collagen type 1.^32,33^ Reduced perfusion in irradiated skin impairs its wound healing potential and complicates reconstructive strategies.

### Fat Grafting

**Fig. 2.**
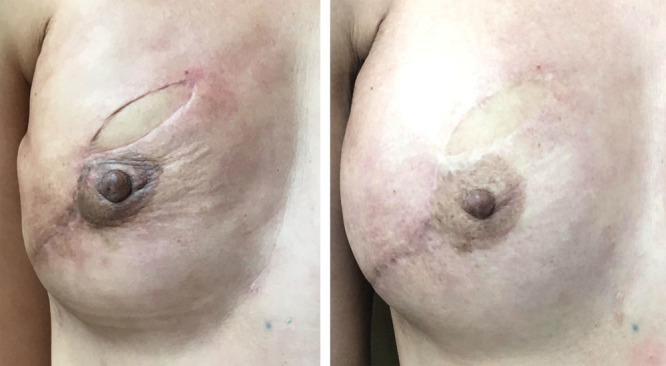
Clinical photograph of a right breast following flap reconstruction and irradiation with clinical signs of texture and pigmentation changes (A). The same breast is shown 12 months after fat grafting demonstrating long-term clinical improvements in contour and skin quality (B).

Treatment of chronic radiodermatitis is lacking, with most current interventions failing to adequately restore skin form and function following radiation-induced damage.^34^ AFG, however, is one emerging modality able to reverse and rejuvenate the fibrotic changes in the skin (Fig. 2). In 2007, Rigotti et al.^8^ first demonstrated the curative effects of AFG in irradiated human skin. They applied purified autologous fat cells to radiation-induced wounds of 20 breast cancer patients. The grafted fat improved clinical symptoms and healed wounds faster with evidence neovascularization. Multiple clinical studies have since confirmed that fat grafting can reverse chronic radiation-induced skin alterations.^35–39^ In 2009, Panettiere et al.^38^ treated irradiated postsurgical breasts with fat grafts and found this to improve patients’ clinical symptoms and clinical scores compared with patients who did not received fat grafts. Grafting of fat into the irradiated skin of breast cancer patients before implant placement, reduced the complications of subsequent breast reconstruction surgery including implant exposure and capsular contracture—which are typically increased in the postirradiated breast.^39^ Additionally, fat grafting also improved skin and subcutaneous tissue quality when delivered at the same time as prostheses in breast reconstructive surgery.^36^ AFG has thus transformed the treatment of radiation-induced skin damage and helped to reconceptualize radiodermatitis as a process that is dynamic and reversible.

Although fat grafting has been used clinically for more than 100 years, the mechanisms by which it exerts its beneficial effects remain incompletely determined. Animal studies have corroborated the clinical findings and helped to elucidate the histological changes underlying these effects. Like in human skin, fat grafted into the irradiated skin of mice improves the healing of radiation-induced ulcers, reduces skin hyperpigmentation, and attenuates dermal thickness and collagen deposition.^20,26,40–42^ The fat grafts also increase skin vascularity, normalize the architecture of skin microvasculature, and enhance the expression of vasculogenic factors including vascular endothelial growth factor (VEGF) and stromal cell-derived factor 1 (SDF-1).^20,26,40,41^

### Role of Adipose-derived Stem Cells

There is increasing support for the idea that the multipotent adipose tissue-derived stromal cells (ASCs) and adipose-derived regenerative cells (ADRCs) within grafted fat are largely responsible for its therapeutic effects.^8^ ASCs are cells with an extensive proliferative capacity and the ability differentiate into multiple mesodermal lineages, including adipocytes, myocytes, chondrocytes, and osteocytes. They comprise up to 3% of the stromal vascular fraction (SVF) of adipose tissue.^43^ The remaining cells of the SVF, collectively referred to as ADRCs, are a heterogenous mix of stem and regenerative cells, including endothelial and smooth muscle progenitors, and preadipocytes.

**Fig. 3.**
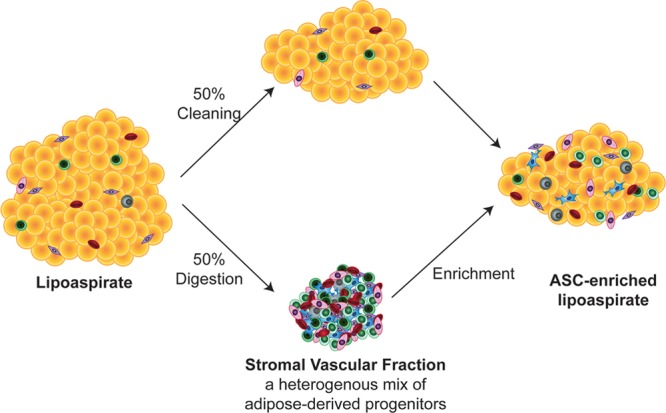
Creation of ASC-enriched fat: A fraction of the fresh lipoaspirate is digested enzymatically to obtain the SVF. The SVF can then be cultured to further enrich for ASCs. The SVF cells or ASCs are then added to the fresh lipoaspirate.

A causative role for ASCs driving the regenerative effects of fat grafting is suggested by their ability to tolerate the hypoxia encountered in the recipient graft site. A significant proportion of grafted fat is resorbed or undergoes necrosis due to the oxidative and ischemic stress. Although mature adipocytes show poor survival upon transplantation, ASCs are able to withstand the ischemia and their proliferative activity is even augmented.^44,45^ Furthermore, a number of studies have demonstrated the superior effects of cell-assisted lipotransfer (CAL) over standard fat grafting. In CAL, fat grafts are enriched with the SVF of the lipoaspirate, or with ASCs isolated from the SVF and expanded in culture (Fig. 3).^46–48^ In both mice and humans, CAL increases the volume of fat retained in the irradiated skin and augments the ability of fat to attenuate radiation-induced increase in dermal thickness.^40,46,49,50^ Additionally, posttransplantation into irradiated graft sites, supplemented fat is of higher quality; it contains fewer cysts and vacuoles and is more vascularized than fat not supplementated.^40,46^

The exact mechanisms by which ASCs rejuvenate the irradiated skin, however, are still unclear. One possibility is that ASCs directly differentiate into adipocytes, endothelial cells, pericytes, and/or smooth muscle cells to regenerate tissue in the recipient site. In the nonirradiated context, cell-tracking experiments have shown that ASCs grafted from genetically labeled green fluorescent protein mice into nonfluorescent recipient mice undergo adipogenic differentiation and incorporation into host adipose tissue.^51^ Other researchers have labeled SVF cells or ASCs with the lipophilic carbocyanine dye, DiI, before transplantation and shown the presence of DiI+ cells among the mature adipocytes in the fat grafted area, which also suggests that the transplanted cells differentiate into adipocytes.^46,52^ Importantly, fibrosis has become increasingly recognized to be intimately involved in adipose tissue dysfunction, and restoration of healthy adipocytes derived from ASCs may contribute to the reversal of fibrosis.^53^ Furthermore, peroxisome proliferator-activated receptor gamma, a master regulator of adipogenesis, has been shown to inhibit profibrotic effects of TGF-β.^54^ However, some research indicates that transplanted ASCs may survive only transiently and that the majority of regenerated tissue may be derived from host cells.^52,55–57^ Furthermore, transcriptional profiling shows that grafted ASCs have low levels of expression of markers for adipogenic differentiation and exhibit no enhanced capacity for adipogenic differentiation than the ASCs within host tissue.^55^

Revascularization posttransplantation is also essential for adipose tissue survival and tissue regeneration. Vasculogenesis is the *de novo* formation and growth of blood vessels from mesodermal derived cells. The SVF is rich in vessel-forming cells such as endothelial cells, pericytes, smooth muscle cells, and their progenitors. These cells, and/or the ASCs, may directly differentiate into vascular cells and assemble into blood vessels. Our laboratory has recently identified 2 progenitor populations from both mouse and human adipose tissue, which are able to form vessels upon transplantation (unpublished data). Other researchers have shown that SVF cells transplanted from green fluorescent protein+ or LacZ+ mice into the adipose tissue of recipient mice create a hybrid vascular network composed of both transplanted ASCs and recipient-derived cells.^58,59^ Likewise, Dil-labeling of ASCs before transplantation also results in Dil+ endothelial cells, indicated by CD31 expression^52^ or expression of von Willebrand factor.^46^ One study reported that the vascular smooth muscle cells in grafted adipose tissue vessels largely originated from the grafted cells, whereas the endothelial cells derived from both graft and host cells. The authors also reported that the host endothelial cells were bone marrow-derived, which suggests that circulating endothelial cells may be mobilized as a result of fat transplantation.^56^

An alternative mechanism by which ASCs mediate their regenerative effects is through paracrine signaling. ASCs have marked proangiogenic effects upon transplantation,^60^ and transcriptional profiling shows that grafted ASCs upregulate their expression of proangiogenic factors.^55^ ASCs secrete multiple potentially synergistic proangiogenic factors, including VEGF, hepatocyte growth factor (HGF), bFGF, and IGF-1.^55,59–62^ The increase in VEGF posttransplantation of ASCs into the mouse fat pads is associated with increased proliferation of neighboring endothelial cells.^57^ The hypoxic conditions in the grafted site may further enhance the secretion of factors such as VEGF and bFGF.^60,63^ Inhibition of apoptosis of proliferating endothelial cells is also critical to allow for the uninhibited growth of new blood vessels. The release of IGF-1 by ASCs has potent antiapoptotic activity and may further promote angiogenesis through this mechanism. Furthermore, IGF-1 also acts as a strong stimulus for preadipocytes to differentiate into mature adipocytes^64,65^ and may help to regenerate the adipose tissue in the grafted site through its pro-adipogenic effects.

The hypoxic environment within the host tissue can also stimulate ASCs to release additional factors with antifibrotic actions. One example is the anti-inflammatory cytokine interleukin (IL)-10, which is released by ASCs,^66^ and has been associated with reduced skin contracture, decreased thickening, and less collagen in upon cell therapy.^67^ Additionally, the ASC-mediated vascularization of the recipient site may lead to decreased tissue fibrosis. Low tissue oxygen levels have been shown to be a strong stimulus for the release of TGF-β1 by resident and infiltrating cells, and this leads to increasing fibrosis with time.^68^ Improved vascularity and, thus oxygenation, may decrease TGF-β1 and its consequent effects on scarring.

**Fig. 4.**
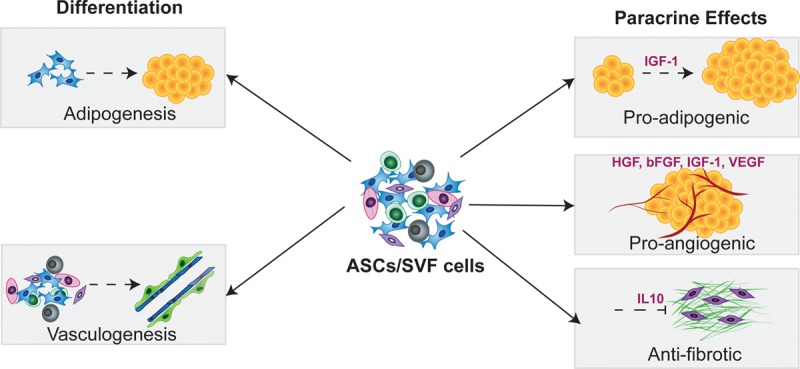
Proposed mechanisms by which ASCs and SVF cells mediate the beneficial effects of fat grafting: ASCs are SVFs thought to help regenerate the tissue in the recipient bed posttransplantation by directly differentiating into adipocytes and vascular cells, as well as by their adipogenic, angiogenic, and anifibrotic paracrine signaling.

In summary, ASCs and SVF cells may act by both differentiation and paracrine signaling to promote revascularization, promote adipogenesis, and reduce apoptosis, which may synergistically regenerate the skin and subcutaneous tissue following radiation-induced injury (Fig. 4).

## CHALLENGES OF FAT GRAFTING INTO IRRADIATED TISSUE

Fat grafting is an emerging treatment, especially in the postirradiated context, and although it is effective at treating radiodermatitis, there are many ongoing considerations. Patients receiving fat grafting post-RT often have severely compromised blood supply in the recipient areas as a result of radiation damage, and this significantly reduces the survival of fat grafts. Methods to improve tissue vascularity before transplantation may, therefore, enhance the survival of grafted fat and, consequently, its regenerative effects. Deferoxamine (DFO) is a Food and Drug Administration (FDA)–approved iron chelator, which has been shown to have angiogenic and antioxidant qualities. DFO stabilizes, and thus increases, levels of hypoxia-inducible factor 1 alpha (HIF-1α) by chelating the iron cofactor of prolyl hydroxylase domain-containing protein 2, the protein which degrades HIF-1α. Increased HIF-1α leads to an increase in downstream angiogenic growth factors^69,70^ and recruitment of endothelial progenitor cells.^71^ In nonirradiated tissue, local DFO treatments improve ischemic flap survival, blood perfusion, and capillary density in animal models.^72,73^ Autologous fat grafts enriched with DFO have been shown to have increased graft survival and viability in rats.^74^ A concern with intragraft injection of DFO, however, is that it may chelate and thus deplete iron, which can impair adipogenesis.^44,45,75^ To circumvent this, preconditioning of irradiated recipient sites with DFO before fat grafting has been performed and we have found that this strategy improved vascularization before grafting, and ultimately enhanced volume of retained fat retained posttransplantation.^76^

Another concern is that fat grafting postirradiation is most often used to regenerate the tissue of patients with an oncologic history. One ongoing concern regarding the use of fat grafting in this context is that transplanted ASCs and SVFs may create an environment conducive to tumor growth or recurrence through their stimulatory paracrine action. Although an in-depth discussion of this is beyond the scope of this review, several *in vitro* and *in vivo* animal studies have suggested a pro-oncologic effect of ASCs/SVFs.^77^ Mature adipocytes can also stimulate the proliferation potential of cancer cells in culture.^78,79^ However, clinical studies have failed to show any increase of breast cancer recurrence after AFG.^80,81^ Furthermore, a number of systematic reviews and meta-analyses have reported no clinical evidence of an increased oncological risk in patients receiving fat grafting. Nonetheless, additional high-quality research is required to investigate this further.^82–85^ As such there is currently no evidence to conclusively support or refute the notion that fat grafting increases oncological risk.

## CONCLUSIONS

RT is an extremely effective oncological therapy but causes severe and long-term collateral damage to tissues, including most often, the skin and subcutaneous tissue. Radiation activates fibrotic pathways in the acute phase, which result in progressive deposition of collagen and substantial dermal induration even years after the initial radiation exposure. The collateral soft-tissue fibrosis can cause significant cosmetic and functional disturbances. AFG is increasingly recognized as a technique able to reverse the radiation-induced fibrosis in the skin. The ASCs and ADRCs within the SVF of grafted fat are thought to be responsible for mediating these beneficial effects, both by direct differentiation and by paracrine signaling. Greater understanding of the mechanisms by which AFG, and the ASCs and ADRCs, rejuvenate the irradiated skin, however, can inform future strategies able to exploit or enhance these effects therapeutically.

## ACKNOWLEDGMENTS

The authors thank the Stanford University Plastic and Reconstructive Surgery Department for providing the biological specimens required for this investigation and the Stanford Small Animal Imaging Facility for use of their imaging instruments and technology. M.T.L. was supported by NIH grants R01 DE026730, R01 DE027323, and R01 GM116892, the Oak Foundation, the Hagey Laboratory for Pediatric Regenerative Medicine, and the Gunn/Olivier Fund. D.C.W. was supported by NIH grants K08 DE024269 and R01 DE027346, the Hagey Laboratory for Pediatric Regenerative Medicine, and the Stanford University Child Health Research Institute Faculty Scholar Award.
